# Bridging the Gap: Missing Data Imputation Methods and Their Effect on Dementia Classification Performance

**DOI:** 10.3390/brainsci15060639

**Published:** 2025-06-13

**Authors:** Federica Aracri, Maria Giovanna Bianco, Andrea Quattrone, Alessia Sarica

**Affiliations:** 1 Department of Medical and Surgical Sciences, Magna Graecia University, 88100 Catanzaro, Italy; federica.aracri@unicz.it (F.A.); mg.bianco@unicz.it (M.G.B.); 2 Neuroscience Research Center, Magna Graecia University, 88100 Catanzaro, Italy; 3 Institute of Neurology, Department of Medical and Surgical Sciences, Magna Graecia University, 88100 Catanzaro, Italy

**Keywords:** imputation, missForest, MICE, Alzheimer’s disease, machine learning

## Abstract

**Background/Objectives:** Missing data is a common challenge in neuroscience and neuroimaging studies, especially in the context of neurodegenerative disorders such as Mild Cognitive Impairment (MCI) and Alzheimer’s Disease (AD). Inadequate handling of missing values can compromise the performance and interpretability of machine learning (ML) models. This study aimed to systematically compare the impacts of five imputation methods on classification performance using multimodal data from the Alzheimer’s Disease Neuroimaging Initiative (ADNI). **Methods:** We analyzed a dataset including clinical, cognitive, and neuroimaging features from ADNI participants diagnosed with MCI or AD. Five imputation techniques—mean, median, k-Nearest Neighbors (kNNs), Multiple Imputation by Chained Equations (MICE), and missForest (MF)—were applied. Classification tasks were performed using Random Forest (RF), Logistic Regression (LR), and Support Vector Machine (SVM). Models were trained on the imputed datasets and evaluated on a test set without missing values. The statistical significance of performance differences was assessed using McNemar’s test. **Results:** On the test set, MICE imputation yielded the highest accuracy for both RF (0.76) and LR (0.81), while SVM performed best with median imputation (0.81). McNemar’s test revealed significant differences between RF and both LR and SVM (*p* < 0.01), but not between LR and SVM. Simpler methods like mean and median performed adequately but were generally outperformed by MICE. The performance of kNNs and MF was less consistent. **Conclusions:** Overall, the choice of imputation method significantly affects classification accuracy. Selecting strategies tailored to both data structure and classifier is essential for robust predictive modeling in clinical neuroscience.

## 1. Introduction

Alzheimer’s Disease (AD) is the most common cause of dementia in the elderly, characterized by the progressive deterioration of memory, language, and reasoning. Mild Cognitive Impairment (MCI) represents an intermediate clinical stage between normal aging and AD, with symptoms that do not yet interfere significantly with daily functioning. Although not all individuals with MCI progress to AD, longitudinal studies suggest that 10–15% progress annually, while others remain cognitively stable. Despite the absence of a definitive cure, current pharmacological treatments may delay symptom progression. Thus, the early identification of MCI patients at high risk for AD is essential to enable timely intervention and disease management [1,2].

Missing data remains a major challenge in neurodegenerative disease research, affecting the reliability and clinical relevance of findings. Despite advances in imputation techniques, key issues persist due to the complexity of multimodal datasets and the intrinsic difficulty of handling incomplete information. A critical obstacle is identifying the underlying mechanism of missingness—whether data are Missing Completely at Random (MCAR), Missing at Random (MAR), or Missing Not at Random (MNAR)—since each scenario demands a specific imputation strategy [3,4,5,6,7,8].

In practice, distinguishing between MAR and MNAR is particularly challenging, often requiring complex modeling and unverifiable assumptions. This uncertainty can introduce bias and compromise inference, especially when inappropriate imputation methods are used. Further complications include high levels of missingness [3], multimodal data integration, cross-cohort variability, longitudinal dropout, and the absence of uncertainty quantification—all of which demand robust, innovative approaches to ensure analytical validity in neurodegenerative disease research.

Large-scale datasets like the Alzheimer’s Disease Neuroimaging Initiative (ADNI) [9] are heavily affected by missing data, with up to 80% of patients presenting incomplete records [10,11]. Since many analytical methods require complete data, researchers are often forced to either discard incomplete entries—at the cost of statistical power and generalizability—or apply imputation techniques. While imputation preserves sample size, it can introduce systematic biases, reduce efficiency, and complicate statistical inference if not properly handled. Moreover, the choice of imputation strategy can significantly influence the performance, interpretability, and explainability of downstream machine learning (ML) models [11,12,13].

The ADNI dataset supports the longitudinal tracking of dementia by capturing structural and functional brain changes across four stages: Cognitively Normal (CN), Significant Memory Concern (SMC), Mild Cognitive Impairment (MCI), and Alzheimer’s Disease (AD) [3,12]. In this study, we compare traditional statistical imputation methods (mean and median) with machine learning-based techniques, including missForest (MF)—a Random Forest-based algorithm [13,14,15], —and the k-Nearest Neighbors imputer (kNNs) [16]. We also evaluate Multivariate Imputation by Chained Equations (MICE) [17,18], a multiple imputation strategy that generates plausible values for missing entries by iteratively modeling each variable [15,19,20]. We assessed the impact of each imputation method on classification performance by predicting dementia stages using the imputed datasets. The methods compared—mean, median, MF, kNNs, and MICE—were evaluated based on their ability to preserve or enhance predictive accuracy. To ensure a comprehensive evaluation across different modeling approaches, we employed three widely used classifiers: Logistic Regression (LR), Random Forest (RF), and Support Vector Machine (SVM). Classification outcomes were measured using standard metrics, including AUC, F1-score, precision, accuracy, and sensitivity.

This study built upon our prior investigations on missing data handling in neurological disorders. In Aracri et al. [13], we conducted a simulation-based study comparing missForest and mean imputation under increasing levels of MCAR, assessing their impacts on the reconstruction of clinical, neuropsychological, and imaging variables in Alzheimer’s Disease data. In Aracri et al. [14], we analyzed the influence of imputation strategies on the performance of tree-based classifiers in Parkinson’s Disease, focusing on classification accuracy and feature relevance. Most recently, in Aracri et al. [15], we compared external versus internal imputation approaches in longitudinal Alzheimer’s Disease data, highlighting how different strategies affect diagnostic model performance over time.

The present study extends this line of research by systematically evaluating how imputation methods influence supervised classification outcomes in dementia staging, using multiple classifiers and statistical tests to assess significance on an independent test set without missing data. Beyond accuracy, we evaluate additional performance metrics—such as sensitivity, precision, and computational efficiency—to provide a more comprehensive comparison across models and imputation strategies. We also analyze feature importance to identify which variables most strongly influence classification outcomes under different imputation conditions. These enhancements offer deeper insight into how data quality and imputation choices affect model robustness and interpretability. This broader perspective not only improves the generalizability of our findings but also offers practical guidance for optimizing machine learning workflows in dementia research. Ultimately, our work contributes to the development of reliable, interpretable, and data-driven classification pipelines for clinical neuroscience applications.

## 2. Materials and Methods

### 2.1. Workflow Analysis

**Figure 1:**
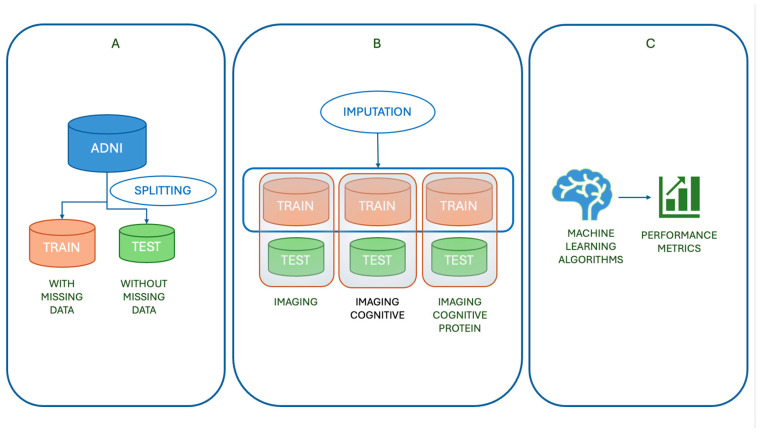
An overview of the study workflow. (**A**) The ADNI dataset is split into a training set (with missing data) and a test set (fully observed). (**B**) Missing data imputation is applied exclusively to the training set using five methods (mean, median, kNNs, MF, MICE) across three incremental data configurations: imaging only, imaging + cognitive, and imaging + cognitive + protein features. The test set remains unchanged throughout. (**C**) The imputed training sets are used to train two machine learning models (Random Forest, Logistic Regression, and Support Vector Machines), whose classification performances are evaluated on the test set using standard metrics.

The study workflow (Figure 1) began with splitting the dataset into training and test sets, with classification performed on both (Figure 1A). Missing data imputation was applied exclusively to the training sets using five methods: mean, median, kNNs, MF, and MICE. The test set remained fully observed. As shown in Figure 1B, the workflow progressed through three stages:

1. **Imaging only:** The initial analysis included only imaging features, with identical columns in both training and test sets. Imputation was applied to the training data before classification.
2. **Imaging + cognitive:** Cognitive variables were added to both datasets, expanding the feature set for classification.
3. **Imaging + cognitive + protein:** Protein biomarkers were incorporated, resulting in a multimodal dataset.

Finally, classification performance was evaluated (Figure 1C) using Random Forest (RF), Logistic Regression (LR) and Support Vector Machines (SVM) trained on the imputed datasets.

All analyses were performed with Python 3.8 and its libraries such as Pandas 2.2, Numpy 2.2, missForest (the python package “missingpy 0.2.0”) and classifier models from SciKit Learn.

### 2.2. Dataset Description and Preparation

We used the ADNIMERGE dataset from the Alzheimer’s Disease Neuroimaging Initiative (ADNI; adni.loni.usc.edu, accessed on 5 July 2022), as described in our previous work [13]. ADNI, launched in 2003 under the leadership of Dr. Michael W. Weiner, integrates MRI, PET, fluid biomarkers, and clinical/neuropsychological assessments to monitor the progression of MCI and early-stage AD [9]. This comprehensive and publicly available dataset offers a robust foundation for investigating imputation strategies in biomedical research.

The version used in this study was downloaded in July 2022 and included baseline data from 738 participants: 336 with Alzheimer’s Disease and 402 with Mild Cognitive Impairment (including both early and late MCI). Unlike our earlier analysis [13], this work incorporated the full dataset, excluding only those variables with ≥80% missing values (Florbetaben [FBB], Pittsburgh Compound B [PIB], AV45, and the Montreal Cognitive Assessment [MOCA]) to avoid bias and preserve analytical robustness.

Table 1 summarizes the demographic, clinical, cognitive, and neuroimaging characteristics of participants with MCI and AD, reported as mean ± standard deviation or frequency. The dataset includes the following:

- **Demographic features**: Age, Sex, and Years of Education (PTEDUCAT);
- **Clinical and cognitive measures**: The Clinical Dementia Rating Sum of Boxes (CDRSB), the Alzheimer’s Disease Assessment Scale (ADAS11, ADAS13, ADASQ4), the Mini-Mental State Examination (MMSE), subtests from the Rey Auditory Verbal Learning Test (RAVLT: immediate recall, learning, forgetting, percent forgetting), Logical Memory–Delayed Recall (LDELTOTAL), Digit Span (DIGITSCOR), the Trail Making Test (TRABSCORE), and the Functional Assessment Questionnaire (FAQ);
- **Genetic information**: Apolipoprotein E (APOE4) genotype;
- **Neuroimaging data**: Glucose metabolism (FDG-PET) and regional brain volumes (Ventricles, Hippocampus, WholeBrain, Entorhinal, Fusiform, Middle Temporal [MidTemp], and Intracranial Volume [ICV]), derived via FreeSurfer.

**Figure 2:**
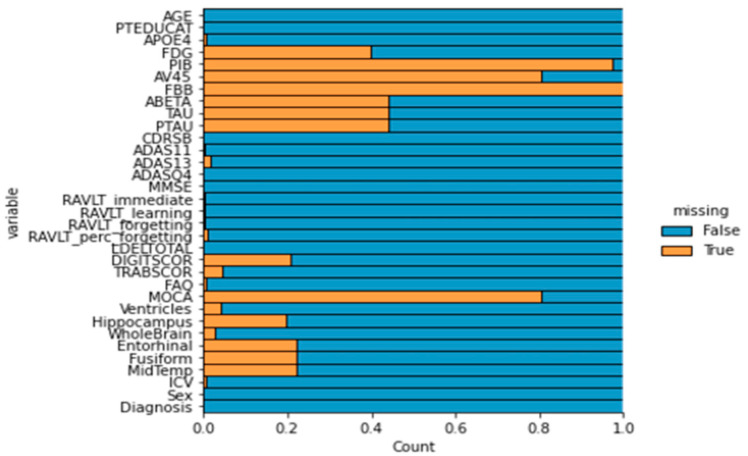
Missing data distribution across all features in the original dataset. The horizontal bars represent the proportion of missing values (in orange) for each variable. Variables with ≥80% missing values, such as PIB, FBB, AV45, and MOCA, were excluded from the final analysis to prevent bias in imputation and model training. Features without missing data are shown entirely in blue.

The extent of missingness across variables is shown in Figure 2. Several features exhibited high proportions of missing data, particularly PET tracers (PIB, FBB, AV45) and the Montreal Cognitive Assessment (MOCA), all of which exceeded the 80% threshold and were excluded from subsequent analyses. Most other variables presented minimal or moderate missingness, making them suitable for imputation and inclusion in the classification workflow.

For analysis purposes, the complete dataset was split into a training set (654 rows, 29 columns) and a test set (84 rows, 29 columns). The test set was fully observed and contained no missing values. As outlined in Figure 1, we evaluated three progressively enriched data configurations:

1. Imaging only: The first configuration included imaging-derived features—glucose metabolism (FDG), Ventricles, Hippocampus, WholeBrain, Entorhinal, Fusiform, and Middle Temporal—and the diagnosis label, resulting in 624 rows and 8 columns in the training set and 84 rows and 8 columns in the test set;
2. Imaging + cognitive: Cognitive variables—Logical Memory–Delayed Recall (LDELTOTAL), Digit Symbol Substitution (DIGITSCOR), and Sex—were added, yielding 11 features for each sample in both sets;
3. Imaging + cognitive + protein: Protein biomarkers (ABETA, TAU, PTAU) were further incorporated, bringing the total to 14 columns for both training (624 rows) and test (84 rows) sets.

### 2.3. Imputation Methods

We evaluated five imputation techniques, ranging from simple statistical approaches to more sophisticated machine learning methods.

Mean and median imputation are basic yet commonly used techniques that replace missing values with the variable’s mean or median, respectively. While easy to implement, these methods assume that observed data adequately represent the underlying distribution and fail to capture inter-variable dependencies, potentially introducing bias, especially in complex or non-normally distributed datasets [20].

k-Nearest Neighbors (kNNs) imputes missing values by identifying the most similar instances based on available features and computing a weighted average from the nearest neighbors [20]. This method is non-parametric, supports mixed data types, and adapts well to non-linear relationships.

Multiple Imputation by Chained Equations (MICE) uses iterative regression modeling to impute missing values multiple times, generating plausible estimates from the observed data. Each incomplete variable is modeled conditionally on the others, allowing for the flexible handling of continuous, categorical, and binary variables [21].

MissForest (MF) is a non-parametric imputation method that uses Random Forest to predict missing values. It iteratively refines estimates using information from other features and supports both categorical and continuous variables. MF also provides internal error estimates via out-of-bag predictions, making it well-suited for high-dimensional biomedical data [13,14,15,22,23,24].

All imputation methods were implemented in Python using default hyperparameters from established libraries:

- SimpleImputer (mean/median) from sklearn.impute, with strategies set to ‘mean’ or ‘median’;
- KNNImputer from sklearn.impute, with n_neighbors = 2;
- IterativeImputer (MICE) from sklearn.impute, with max_iter = 100 and random_state = 42;
- MissForest from the missingpy package (v0.2.0), with criterion = ‘squared_error’, max_features = 1.0, and oob_score = True.

### 2.4. Machine Learning Classification

After imputation, we applied three classification algorithms to distinguish between Alzheimer’s Disease (AD) and Mild Cognitive Impairment (MCI): Logistic Regression (LR), Random Forest (RF) and Support Vector Machine (SVM).

Logistic Regression (LR) is a generalized linear model commonly used for binary classification. It estimates the probability of class membership by modeling the log-odds of the outcome as a linear combination of the input features. LR is widely adopted due to its interpretability, especially via odds ratios, but it assumes a linear relationship between predictors and the log-odds of the target variable [25,26].

Random Forest (RF) is an ensemble learning method based on the aggregation of multiple decision trees trained on bootstrapped samples. It handles non-linear relationships, mixed data types, and missing values effectively. Model performance is internally estimated using out-of-bag (OOB) samples, which reduces the need for external validation sets [1,27]. RF is also robust to overfitting and performs well in high-dimensional settings.

Support Vector Machine (SVM) [28] is a supervised learning algorithm that constructs an optimal hyperplane to separate data points belonging to different classes by maximizing the margin between them. It is particularly effective in high-dimensional spaces and can be adapted to non-linear classification problems through the use of kernel functions.

These three models were selected to represent distinct types of learning algorithms, a linear model (LR), a non-linear ensemble method (RF), and a margin-based classifier (SVM), enabling a comprehensive comparison of imputation strategies across different model architectures.

### 2.5. Performance Evaluation

#### 2.5.1. Training Set

To evaluate model performance under different imputation strategies, we applied a stratified 5-fold cross-validation on the training data. This method preserved the class distribution across folds and mitigated the risk of overfitting. Each imputation method was independently applied to the training set, enabling a systematic comparison of their impact on classification accuracy and robustness. This procedure also helped to identify potential biases introduced by the imputation process and to assess the generalizability of the models.

#### 2.5.2. Testing Set

Classifier performance was assessed on a held-out test set, using the same features included in the corresponding training configurations. To quantify discriminatory power, we computed the Area Under the Receiver Operating Characteristic Curve (AUC-ROC), where higher values indicated a better ability to distinguish between MCI and AD. This consistent evaluation framework enabled a direct comparison across imputation strategies while isolating their influence on classification outcomes. Additional performance metrics computed on the test set included the following:

- Accuracy: The proportion of correctly predicted instances over the total;
- Precision: The proportion of true positives among all predicted positives;
- Recall (Sensitivity): The proportion of actual positives correctly identified;
- F1 Score: The harmonic mean of precision and recall, balancing both in a single metric—especially relevant in imbalanced settings.

To assess whether the differences in classification performance between the imputation strategies were statistically significant, we applied the McNemar test. This non-parametric test is suitable for comparing the prediction outcomes of two classifiers evaluated on the same test set, particularly in binary classification scenarios. The McNemar test was then applied to determine whether the observed differences in misclassification patterns were statistically significant (*p* < 0.05).

## 3. Results

**Figure 3:**
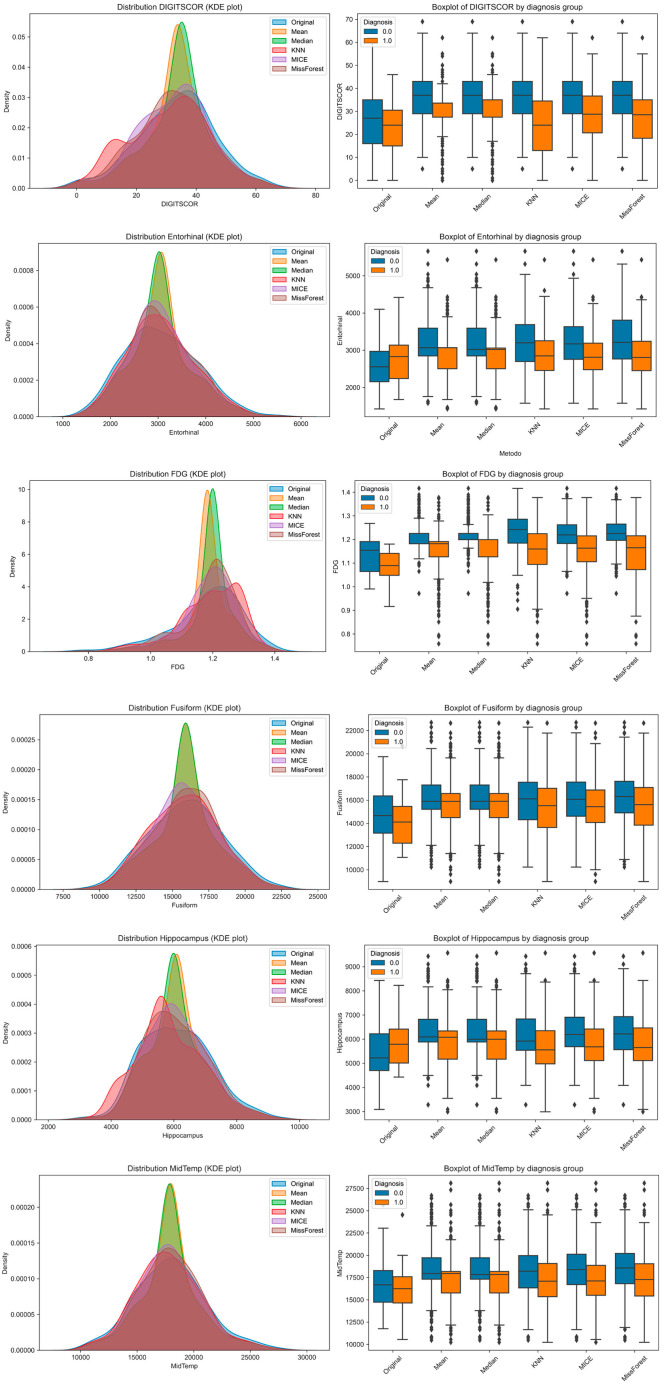
The impacts of different imputation methods on the distribution and diagnostic separability of selected features. Left column: Kernel Density Estimation (KDE) plots of the variable distributions before (Original) and after imputation using five methods: mean, median, k-Nearest Neighbors (kNNs), Multiple Imputation by Chained Equations (MICE), and missForest. Right column: Boxplots of the same variables stratified by diagnosis group (0 = MCI, 1 = AD), illustrating group-level differences and distributional shifts across imputation strategies. Features include DIGITSCOR (neuropsychological), Entorhinal volume, FDG uptake, Fusiform volume, Hippocampal volume, and Middle Temporal volume.

To explore how imputation methods affect the distributional properties of individual features and their separability across diagnostic groups, we visualized six representative variables using Kernel Density Estimation (KDE) plots and group-stratified boxplots (Figure 3). These included one cognitive variable (DIGITSCOR), one PET-based metabolic marker (FDG), and four structural MRI-derived regional volumes (Entorhinal, Fusiform, Hippocampus, and Middle Temporal cortex). KDE plots allowed for performing a direct comparison between the original (complete) distribution and those resulting from each imputation method. Boxplots, stratified by diagnosis (MCI vs. AD), illustrate the extent to which group differences are preserved or distorted following imputation.

Notably, while all methods approximated the original distributions to varying degrees, missForest and MICE produced distributions more consistent with the original data. However, group separability was occasionally reduced, particularly with mean and median imputation, suggesting potential limitations in preserving clinically relevant signals.

Classification performance on the training set, averaged over five cross-validation folds, is reported in Table 2 (Random Forest), Table 3 (Logistic Regression), and Table 4 (SVM). For Random Forest (Table 2), mean imputation achieved the best accuracy (0.80 ± 0.033) and F1-score (0.78 ± 0.035), while median imputation provided a slightly lower but comparable performance. MICE yielded the lowest results across all metrics, suggesting that it may not be well-suited to RF in this dataset. Interestingly, missForest and kNNs did not perform as strongly as expected, with MF performing better than kNNs but still below mean and median. In the case of Logistic Regression (Table 3), kNN imputation led to the highest performances across all metrics—accuracy (0.73 ± 0.038), precision (0.74 ± 0.05), recall (0.67 ± 0.082), and F1-score (0.70 ± 0.058)—indicating good compatibility with linear models in terms of signal preservation. Mean, median, and MICE imputations produced comparable but slightly lower results, while MF remained consistent but not superior.

For SVM (Table 4), multiple imputation methods resulted in very similar accuracies (~0.72) and F1-scores (~0.69–0.70), with minor variations across imputation strategies. MICE yielded the highest recall (0.69 ± 0.091) and a balanced profile across all metrics. Interestingly, missForest and kNNs showed stronger performance in SVM than in RF, again confirming that the effectiveness of imputation methods is model-dependent. Overall, these training results emphasize the interaction between classifier type and imputation strategy. While mean imputation appeared most effective for Random Forest, kNNs was best suited to Logistic Regression, and MICE performed robustly across all metrics in SVM. This reinforces the importance of jointly considering both imputation and model choice during pipeline design.

The classification performances of the three models—Random Forest (RF), Logistic Regression (LR), and Support Vector Machine (SVM)—on the test set across different imputation methods are reported in Table 5, Table 6 and Table 7. For RF, the highest F1-score (0.70) and recall (0.79) were obtained with MICE imputation, while mean imputation yielded the highest precision (0.65). Notably, kNNs and missForest consistently underperformed across all metrics in the RF model, suggesting reduced compatibility with ensemble-based classification in this setting. In the case of LR, MICE again provided the best overall performance, achieving the highest values for accuracy (0.81), precision (0.71), and F1-score (0.73), along with a strong recall (0.76). Mean and median imputations also performed well, whereas kNN imputation led to a substantial drop in recall (0.48) and F1-score (0.53), indicating poor sensitivity. SVM yielded the most robust recall (0.83) when combined with MICE, while median imputation resulted in the highest accuracy (0.81) and F1-score (0.74). All imputation methods except kNNs produced relatively balanced precision and recall values, reinforcing the observation that kNN imputation tends to impair classifier performance in this dataset. Overall, MICE emerged as the most consistent imputation strategy across models, particularly in preserving sensitivity (recall) and optimizing F1-score, which is especially relevant in clinical applications where minimizing false negatives is critical. Conversely, kNNs and missForest showed less stable behavior, highlighting the importance of tailoring imputation approaches to the classifier architecture and dataset characteristics.

To assess whether the differences in classification outcomes among models were statistically significant, we applied McNemar’s test to pairwise comparisons between classifiers on the test set (Table 8). The results show that the differences between Random Forest (RF) and both Logistic Regression (LR) and Support Vector Machine (SVM) were statistically significant, with *p*-values of 0.0059 and 0.0012, respectively. This indicates that RF produced a significantly different pattern of misclassifications compared to LR and SVM. In contrast, the comparison between LR and SVM yielded a McNemar statistic of 0.00 and a *p*-value of 1.00, suggesting no significant difference in their classification decisions. These findings support the superior and distinct behavior of RF in this context, while LR and SVM appear to behave similarly in terms of misclassification patterns.

**Figure 4:**
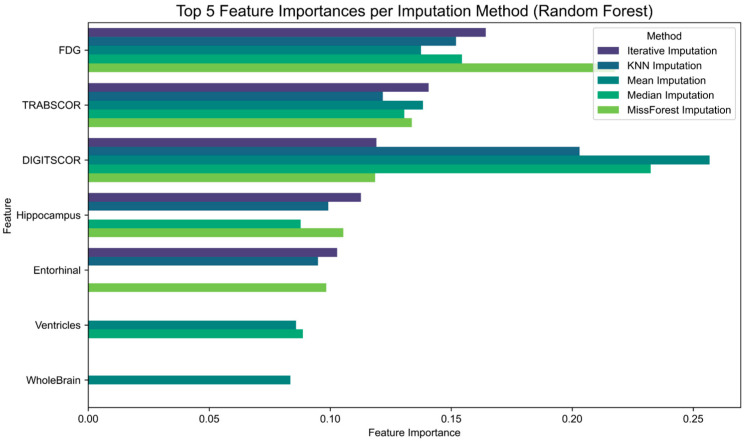
Feature importance scores across imputation methods based on Random Forest classification. Each horizontal bar represents the relative importance of a feature for predicting diagnostic labels (MCI vs. AD), as computed by Random Forest classifiers trained on datasets imputed using different strategies: mean, median, k-Nearest Neighbors (kNNs), Multiple Imputation by Chained Equations (Iterative), and missForest. The figure highlights how the imputation method used can influence the selection and ranking of predictive features, potentially affecting model interpretability and clinical relevance.

Finally, to assess the impact of imputation strategies on feature relevance, we examined the feature importance scores derived from Random Forest classifiers trained on each imputed dataset (Figure 4). The results show that although some features consistently rank among the most informative across imputation methods, their relative importance varies depending on the technique applied. For instance, hippocampal volume and FDG uptake maintain high relevance across all conditions, while other features such as entorhinal cortex or cognitive scores exhibit greater variability.

## 4. Discussion

This study systematically investigated the impacts of five imputation methods—mean, median, k-Nearest Neighbors (kNNs), Multiple Imputation by Chained Equations (MICE), and missForest (MF)—on the classification of Alzheimer’s Disease (AD) and Mild Cognitive Impairment (MCI) using multimodal data from the ADNI cohort. By evaluating the performances of three classification models—Random Forest (RF), Logistic Regression (LR), and Support Vector Machine (SVM)—on an independent test set without missing data, we demonstrated that the choice of imputation strategy can significantly influence classification outcomes. Our findings emphasize that this impact varies not only across imputation techniques but also depending on the classifier used.

Among the evaluated imputation methods, MICE consistently yielded the highest classification accuracy with both RF and LR, while SVM performed best with median imputation. Although MF and kNNs have been reported in the literature as robust choices for mixed-type data, in our experiments, their performances were less stable and did not generalize as effectively across classifiers. McNemar’s test confirmed that RF differed significantly from both LR and SVM in terms of misclassification patterns, reinforcing the importance of matching imputation strategies to the model architecture.

Interestingly, simpler methods such as mean and median imputation delivered competitive performances, particularly when combined with RF. This suggests that in structured datasets like the ADNI, model capacity can partially compensate for the loss of information due to simplistic imputation. Nevertheless, these statistical approaches remain limited by their inability to preserve inter-variable relationships and may lead to biased estimates when missingness is not completely random.

Our findings are consistent with previous work by Aracri et al. [13,14,15] and align with the recent literature [20,29], confirming the effectiveness of ML-based imputation methods—particularly missForest—in handling missing data within complex biomedical datasets. In prior studies, these approaches demonstrated improved classification performance in both cross-sectional and longitudinal settings, especially in predicting cognitive decline, modeling disease progression, and addressing class imbalance in multiclass scenarios such as Parkinson’s Disease and SWEDD [14]. In our current work, we further confirm that ML-based imputation methods offer clear advantages over traditional statistical techniques in preserving inter-variable relationships and maintaining classification accuracy across multiple classifiers. Although all tested imputation methods produced acceptable results on a clean test set, ML-based techniques more consistently captured nuanced patterns and preserved clinically meaningful information—qualities that are crucial in real-world, heterogeneous datasets. These results highlight the importance of selecting imputation strategies that are not only statistically valid but also tailored to the structure, complexity, and intended analytical use of the data.

Clinically, the ability of MF and kNNs to maintain high sensitivity suggests their potential for reducing false negatives in diagnostic systems—an essential requirement for timely intervention in prodromal Alzheimer’s Disease. Conversely, the higher precision achieved by simpler imputation methods may be advantageous in contexts where false positives entail significant clinical or resource-related consequences. These trade-offs underscore the importance of aligning imputation and classification strategies with the specific objectives and risk profiles of clinical applications.

Beyond conventional imputation approaches, emerging deep learning-based methods offer new opportunities to enhance this balance. In particular, Generative Adversarial Networks for Synthetic Oversampling (GANSOs) [30] have been proposed as a powerful tool for imputing missing values by modeling complex joint data distributions and generating realistic synthetic samples. This technique has shown promising results in psychiatric research, where datasets are frequently characterized by high dimensionality, small sample sizes, and non-random missingness—conditions also present in neurodegenerative disease studies.

While this study focused on Alzheimer’s Disease (AD) and Mild Cognitive Impairment (MCI), the proposed approach is generalizable to other neurodegenerative conditions and datasets that integrate neuroimaging, cognitive, and biomarker data [31]. The imputation classification workflow that we present can be readily extended to more complex analytical tasks, such as multimodal data fusion, survival analysis, or uncertainty-aware AI systems—contexts in which imputation quality has a direct impact on both model interpretability and the reliability of clinical decision support.

Despite its strengths, this study presents several limitations that warrant consideration. First, we assumed a Missing at Random (MAR) mechanism without explicitly testing or modeling the missingness process. However, real-world clinical datasets often include values that are Missing Not at Random (MNAR), which would require more sophisticated techniques to avoid bias and ensure validity. Second, imputation was performed externally prior to model training. While this approach avoids information leakage into the test set, it does not consider potential interactions between imputation and classification. Future work could explore joint or end-to-end frameworks that integrate both steps to better reflect real-world workflows. Third, all imputation techniques were applied using default hyperparameters. Although this choice improved reproducibility and facilitates comparison, it may have limited the performance of algorithms such as missForest and kNNs, which are known to be sensitive to parameter tuning. Optimization strategies such as grid or randomized searches could enhance these methods and should be considered in future studies. Fourth, the analysis was restricted to baseline cross-sectional data. Longitudinal validation is essential to assess the temporal stability and clinical robustness of imputation methods, particularly in applications involving disease progression modeling or treatment response monitoring. Finally, an important yet underexplored issue concerns the estimation of the minimum sample size required to achieve reliable classification performance in the presence of missing data. In clinical research, where small samples and incomplete data often coexist, understanding this relationship is crucial. Salazar et al. [32] recently proposed a proxy learning curve method based on the Bayes classifier to estimate the minimum number of samples needed to reach a desired classification accuracy. Although originally developed for complete data, this theoretical framework could be extended to imputed datasets. Integrating such approaches into future research would help to quantify the interplay between sample size, missingness, and model robustness in biomedical machine learning.

## 5. Conclusions

This study systematically evaluated the impacts of five imputation methods—mean, median, k-Nearest Neighbors (kNNs), Multiple Imputation by Chained Equations (MICE), and missForest (MF)—on the classification of Mild Cognitive Impairment (MCI) and Alzheimer’s Disease (AD) using multimodal data from the ADNI cohort. Classification was performed using three machine learning models: Random Forest (RF), Logistic Regression (LR), and Support Vector Machine (SVM). All models were trained on imputed datasets and tested on a clean, independent test set without missing values to ensure unbiased performance assessment.

Our findings show that MICE consistently yielded the highest classification accuracy with RF and LR, while SVM achieved its best performance with median imputation. McNemar’s test revealed significant differences in misclassification patterns between RF and the other classifiers, highlighting how model architecture interacts with the chosen imputation method. Although simple statistical imputations (mean, median) provided acceptable results, more sophisticated approaches like MICE demonstrated clear advantages in maintaining classification accuracy and generalizability.

Furthermore, the analysis of feature importance revealed that certain features retained their discriminative power across imputation methods, while others were more sensitive to the chosen strategy, underscoring the influence of imputation not only on overall performance but also on model interpretability.

These results emphasize the need to select imputation strategies that are tailored to both the data structure and the model in use. Future research should consider the joint optimization of imputation and classification workflows, explore deep learning-based imputation techniques, and extend validation to longitudinal datasets and real-world clinical settings. Such integration is essential for developing robust, interpretable, and reproducible machine learning pipelines in the context of neurodegenerative disease diagnostics.

## Figures and Tables

**Table 1:** Descriptive statistics (mean ± standard deviation or frequency) of features derived by the diagnosis of Mild Cognitive Impairment (MCI) and Alzheimer’s Disease (AD) as in the complete dataset.

	MCI (*n* = 164)	AD (*n* = 64)
AGE	74.28 ± 6.97	75.48 ± 7.01
SEX (M/F)	112/52	38/26
PTEDUCAT (years)	15.77 ± 2.87	14.70 ± 3.18
APOE4 (0/1/2)	69/72/23	22/34/8
FDG	1.24 ± 0.08	1.15 ± 0.09
CDRSB	1.49 ± 0.74	4.59 ± 1.75
ADAS11	10.85 ± 4.23	18.52 ± 5.82
ADAS13	17.79 ± 6.11	28.93 ± 6.96
ADASQ4	6 ± 2.27	8.59 ± 1.56
MMSE	27.19 ± 1.72	23.36 ± 2.09
RAVLT immediate	31.37 ± 8.89	22.94 ± 6.28
RAVLT learning	3.40 ± 2.43	1.5 ± 1.50
RAVLT forgetting	4.72 ± 2.15	4.48 ± 1.60
RAVLT perc forgetting	67.21 ± 30.64	89.91 ± 18.55
LDELTOTAL	4.04 ± 2.7	1.08 ± 1.92
DIGITSCOR	37.73 ± 10.77	26.12 ± 10.38
TRABSCOR	130.05 ± 72.52	209.17 ± 82.26
FAQ	3.54 ± 3.95	13.41 ± 6.39
Ventricles	43,035.42 ± 23,935.51	50,807.48 ± 19,979.86
Hippocampus	6515.82 ± 1.081.29	5595.41 ± 959.16
WholeBrain	1.01 × 10^9^ ± 106,289.37	957,480.62 ± 91,592.30
Entorhinal	3357.73 ± 754.65	2738.62 ± 687.03
Fusiform	16,740.73 ± 2253.76	15,180.72 ± 2381.71
MidTemp	18,962.59 ± 2680.51	16,896.44 ± 2926.67
ICV	1.58 × 10^9^ ± 162,490.76	1.56 × 10^9^ ± 150,093.89

Abbreviation: MCI = Mild Cognitive Impairment; AD = Alzheimer Disease; PTEDUCAT = Education; APOE4 = genotype (subgroups analysis); FDG = Rates of change in glucose metabolism (average FDG-PET of angular, temporal, and posterior cingulate); CDRSB = Rate of Decline; ADAS11, ADAS13, ADASQ4 = Neuropsychometric Testing Supplies; MMSE = Cognitive Screening Test; RAVLT = Memory Test; LDELTOTAL = Logical Memory–Delayed Recall; DIGITSCOR = Digit Symbol Substitution (working memory); TRABSCOR = Trail Making Test; FAQ = Functional Assessment Questionnaire (Activities of Daily Living); MIDTEMP = Middle Temporal; Ventricles, Hippocampus, WholeBrain, Entorhinal, Fusiform = Rate of volume change in structural MRI measures; ICV = Intracranial Total Volume.

**Table 2:** The classification performances of the Random Forest model on the training set using different imputation methods. Metrics are reported as mean ± standard deviation across five cross-validation folds.

Random Forest	Accuracy	Precision	Recall	F1 Score
Mean Imputation	**0.80 ± 0.033**	**0.80 ± 0.052**	**0.76 ± 0.035**	**0.78 ± 0.035**
Median Imputation	0.79 ± 0.029	0.79 ± 0.037	0.75 ± 0.040	0.77 ± 0.031
kNN Imputation	0.74 ± 0.038	0.75 ± 0.055	0.68 ± 0.053	0.71 ± 0.041
MICE Imputation	0.70 ± 0.039	0.69 ± 0.048	0.66 ± 0.050	0.67 ± 0.044
MF Imputation	0.76 ± 0.040	0.76 ± 0.057	0.72 ± 0.044	0.74 ± 0.042

The best results are shown in bold.

**Table 3:** The classification performances of the Logistic Regression model on the training set using different imputation methods. Metrics are reported as mean ± standard deviation across five cross-validation folds.

Logistic Regression	Accuracy	Precision	Recall	F1 Score
Mean Imputation	0.72 ± 0.05	0.73 ± 0.069	0.65 ± 0.51	0.69 ± 0.055
Median Imputation	0.71 ± 0.047	0.72 ± 0.071	0.63 ± 0.044	0.67 ± 0.051
kNN Imputation	**0.73 ± 0.038**	**0.74 ± 0.05**	**0.67 ± 0.082**	**0.70 ± 0.053**
MICE Imputation	0.72 ± 0.046	0.73 ± 0.059	0.66 ± 0.055	0.69 ± 0.054
MF Imputation	0.73 ± 0.041	0.74 ± 0.056	0.65 ± 0.040	0.69 ± 0.045

The best results are shown in bold.

**Table 4:** The classification performances of SVM model on the training set using different imputation methods. Metrics are reported as mean ± standard deviation across five cross-validation folds.

SVM	Accuracy	Precision	Recall	F1 Score
Mean Imputation	0.71 ± 0.057	0.69 ± 0.059	0.69 ± 0.085	0.69 ± 0.069
Median Imputation	0.70 ± 0.057	0.70 ± 0.065	0.66 ± 0.092	0.67 ± 0.072
kNN Imputation	0.72 ± 0.051	0.71 ± 0.055	0.70 ± 0.073	0.70 ± 0.059
MICE Imputation	**0.72 ± 0.053**	**0.71 ± 0.052**	**0.69 ± 0.091**	**0.70 ± 0.069**
MF Imputation	0.72 ± 0.060	0.71 ± 0.058	0.68 ± 0.094	0.69 ± 0.075

The best results are shown in bold.

**Table 5:** The classification performances of the Random Forest model on the test set using different imputation methods.

Random Forest	Accuracy	Precision	Recall	F1 Score
Mean Imputation	0.75	0.65	0.59	0.62
Median Imputation	0.71	0.59	0.59	0.59
kNN Imputation	0.69	0.55	0.52	0.54
MICE Imputation	**0.76**	**0.62**	**0.79**	**0.70**
MF Imputation	0.67	0.52	0.55	0.53

The best results are shown in bold.

**Table 6:** The classification performances of the Logistic Regression model on the test set using different imputation methods.

Logistic Regression	Accuracy	Precision	Recall	F1 Score
Mean Imputation	0.80	0.70	0.72	0.71
Median Imputation	0.80	0.69	0.76	0.72
kNN Imputation	0.70	0.58	0.48	0.53
MICE Imputation	**0.81**	**0.71**	**0.76**	**0.73**
MF Imputation	0.79	0.68	0.74	0.70

The best results are shown in bold.

**Table 7:** The classification performances of the SVM model on the test set using different imputation methods.

SVM	Accuracy	Precision	Recall	F1 Score
Mean Imputation	0.80	0.69	0.76	0.71
Median Imputation	**0.81**	**0.70**	**0.79**	**0.74**
kNN Imputation	0.75	0.64	0.62	0.63
MICE Imputation	0.79	0.65	0.83	0.73
MF Imputation	0.80	0.69	0.76	0.72

The best results are shown in bold.

**Table 8:** The table reports McNemar statistics and corresponding *p*-values for comparisons between Random Forest (RF), Logistic Regression (LR), and Support Vector Machine (SVM) based on their classification outcomes on the test set. Statistically significant results (*p* < 0.05) are marked with an asterisk (*).

ML Models Comparison	McNemar Statistic	*p*-Value
RF vs. LR	7.58	0.0059 *
RF vs. SVM	10.56	0.0012 *
LR vs. SVM	0.00	1.00

## Data Availability

Data used in preparation of this article were obtained from the Alzheimer’s Disease Neuroimaging Initiative (ADNI) database (adni.loni.usc.edu, accessed on 5 July 2022).

## Abbreviations

The following abbreviations are used in this manuscript:

MCI	Mild Cognitive Impairment
AD	Alzheimer’s Disease
ADNI	Alzheimer’s Disease Neuroimaging Initiative
ML	Machine Learning algorithms
CN	Cognitively Normal
SMC	Significant Memory Concern
MF	MissForest algorithm
kNN	k-Nearest Neighbors imputationi
MICE	Multivariate Imputation by Chained Equations
MAE	Mean Absolute Error
RMSE	Root Mean Square Error
NRMSE	Normalized Root Mean Square Error
MRI	Magnetic Resonance Imaging
PET	Positron Emission Tomography
AUC	Area Under the Curve
LR	Logistic Regression
RF	Random Forest
TPR	True Positive Rate
FPR	False Positive Rate
